# Development, assessment and educational impact of a blended e-learning training program on pharmacovigilance implemented in four African countries

**DOI:** 10.3389/fmed.2024.1347317

**Published:** 2024-04-17

**Authors:** Francesco Schievano, Kissa W. Mwamwitwa, Seth Kisenge, Elice Mmari, Alemayehu Duga, Siphesihle Nhlabatsi, Cassandra Elagbaje, Abiodun Sadikat Abiola, Solomon Getnet Meshesha, Silvia Pagani, Riccardo Lora, Alberto Sabaini, Frank Cobelens, Linda Härmark, Eric Battey Eko, Anita Conforti, Mauro Venegoni, Lara Magro, Ugo Moretti

**Affiliations:** ^1^Section of Pharmacology, Department of Diagnostics and Public Health, University of Verona, Verona, Italy; ^2^Section of Clinical Trials Control and Pharmacovigilance, Tanzania Medicines and Medical Devices Authority (TMDA), Dar es Salaam, Tanzania; ^3^KNCV Tuberculosis Foundation, Dar es Salaam, Tanzania; ^4^Doctoral School Societies, Politics, Public Health, Pharmacoepidemiology and Pharmacovigilance, University of Bordeaux, Bordeaux, France; ^5^National Pharmacovigilance Center, Eswatini Ministry of Health, Mbabane, Eswatini; ^6^Baylor College of Medicine Children’s Foundation-Eswatini, Mbabane, Eswatini; ^7^KNCV Tuberculosis Foundation, Abuja, Nigeria; ^8^National Agency for Food and Drug Administration and Control (NAFDAC), Abuja, Nigeria; ^9^Armauer Hansen Research Institute (AHRI), Addis Ababa, Ethiopia; ^10^Unit of Medicine, Hospital of Vimercate, Vimercate, Italy; ^11^MedBrains, Department of Computer Science, University of Verona, Verona, Italy; ^12^Department of Global Health and Amsterdam Institute for Global Health and Development, Amsterdam University Medical Centers, University of Amsterdam, Amsterdam, Netherlands; ^13^Netherlands Pharmacovigilance Centre Lareb, ‘s-Hertogenbosch, Netherlands

**Keywords:** pharmacovigilance, blended-learning, tuberculosis, cascade training, Africa

## Abstract

**Introduction:**

Efforts to improve medication access in low-and middle-income countries, particularly in Sub-Saharan Africa, have made progress, especially in the fight against infectious diseases such as tuberculosis. However, challenges exist in establishing effective pharmacovigilance systems. The PhArmacoVIgilance Africa (PAVIA) project was committed to enhancing pharmacovigilance in Tanzania, Eswatini, Nigeria, and Ethiopia, with an emphasis on anti-tuberculosis drugs, utilizing various methods, including training. This study evaluates the PAVIA training program’s effectiveness and its adaptation during the COVID-19 pandemic.

**Methods:**

A blended e-learning program, incorporating two courses and a platform for educational materials, was developed. This program, designed to train healthcare professionals in pharmacovigilance, was incorporated into a Training of Trainers model. To evaluate the program effectiveness, we used multiple measures such as assessing knowledge gain through pre-and post-test scores, assessing learners’ satisfaction and attitudes via questionnaires, and analyzing Individual Case Safety Reports (ICSRs) in VigiBase to determine the impact on spontaneous reporting systems in the PAVIA countries.

**Results:**

121 learners enrolled in the pilot trainings, including 36 from Tanzania, 34 from Eswatini, 25 from Nigeria, and 26 from Ethiopia. Notably, post-test scores were significantly higher than pre-test scores in all four countries. Following the pilot trainings, multiple step-down training sessions were held in Tanzania, Eswatini, and Nigeria, with a total of 827 learners registering and 421 successfully completing the program. Learners’ scores on the post-tests were significantly higher than on the pre-tests for both courses in all three countries. Learners’ feedback on the training was overwhelmingly positive. Additionally, a qualitative analysis of ICSRs revealed a substantial increase in reports after the training in Tanzania, Eswatini, and Nigeria.

**Discussion:**

An innovative e-learning program trained healthcare professionals in pharmacovigilance and anti-tuberculosis drug safety over 3 years in four PAVIA countries. The program effectively improved participants’ knowledge, received positive feedback, and likely had an impact on reporting rates in Tanzania, Eswatini, and Nigeria, although a direct causal link could not be definitively established due to data limitations and other factors, such as the heightened reporting rates associated with COVID-19 vaccines, that could have contributed to the notable increase in ICSRs.

## Introduction

In recent years, the World Health Organization (WHO) has worked relentlessly in close collaboration with international partners, research institutions, donors and partners from the private sector to improve global accessibility and availability of medicines, vaccines and other health products (1). Evidence shows that there has been a notable increase in the availability and utilization of a wide range of essential medications in low-and middle-income countries (LMICs) (2). This has been especially true for sub-Saharan Africa (SSA) countries, which bear the highest burden of diseases worldwide, where people have strongly benefitted from the expanded access to newer and established medicines against infectious conditions such as AIDS/HIV, malaria and tuberculosis (TB). In 2015 bedaquiline, a newly launched drug for the treatment of drug-resistant tuberculosis (DR-TB), was included in the TB treatment programs in South Africa and Tanzania (3, 4). Delamanid, another recently approved anti-TB drug for DR-TB, was first recommended in 2014 by the WHO (5). Since then delamanid has been used under different conditions in a number of SSA countries, including Tanzania, Eswatini, Nigeria and Ethiopia (6). The most recent drug for the treatment of highly DR-TB is pretomanid, which is used in combination with bedaquiline and linezolid (7). Pretomanid has been already approved or is undergoing the approval process in various SSA countries, including Ethiopia and Nigeria, where regulatory approval submission is underway (8).

As the number of individuals who gain access to new and better therapeutics keeps growing at a fast pace in LMICs, so does the need for monitoring drug safety through accurate pharmacovigilance (PV) processes.

Although every single phase of the life cycle of a drug, including the so-called pre-marketing phase, requires attentive monitoring of drug safety, post-marketing safety surveillance of drugs acquires particular relevance as conditions of drug use are different from those commonly seen during the pre-marketing phase. Before reaching the market, medicines go through a developing process that includes multiple clinical trials, during which their efficacy and safety are tested and monitored under controlled conditions for a relatively limited length of time. Such conditions cannot be replicated in a real-world setting, where the same medicines become widely available to a huge number of different subpopulations (9). At this point, post-marketing PV comes into play (10).

To ensure the effective surveillance of drug safety and the timely detection of potential issues that may arise from their use, maintaining a thorough and robust PV system is imperative. Operating a well-functioning PV system needs financial and technical resources which are often deficient in SSA countries (11). A comprehensive assessment of PV systems of SSA countries conducted in 2011 found that 87% of such countries did not have a functional PV system. In contrast, only 4 out of 46 SSA countries could rely on a performing PV system capable of detecting, evaluating, and preventing medicine safety issues (12).

PhArmacoVIgilance Africa (PAVIA) was a consortium comprising 13 international partners operational from 2018 to 2023, funded by the European Developing Countries Clinical Trials Partnership (EDCTP). The PAVIA Project, led by the Amsterdam Institute for Global Health and Development (AIGHD), had a primary goal of enhancing drug safety monitoring and strengthening PV processes in four SSA countries: Ethiopia, Eswatini, Nigeria, and Tanzania. It included five Work Packages.^1^ The project placed particular emphasis on actively surveilling the safety of anti-TB drugs, a responsibility carried out by the National Tuberculosis Programs (NTPs).

When examining the healthcare landscape in African countries, literature consistently points out a set of enduring challenges. These encompass the shortage of skilled healthcare professionals (HCPs) and their inadequate retention, underdeveloped healthcare infrastructure, low levels of PV awareness among HCPs, and the vulnerability of regulatory and legal frameworks. Notably, one of the most formidable hurdles to PV progress in resource-limited environments is the shortage of personnel trained in PV (13, 14).

In 2018, the PAVIA team conducted a baseline assessment of PV systems and NTPs in Eswatini, Ethiopia, Nigeria, and Tanzania. The strengths and challenges of these systems were assessed, with a focus on their capacity to monitor safety of medicines registered and not registered by the National Medicines Regulatory Authorities (NMRAs) for the treatment of DR-TB. The assessment revealed that all countries had a national health policy in place. A national drug policy was available in three of the four countries, but as part of the health policy in Ethiopia. All four countries had spontaneous reporting systems in place, although these received few reports. Eswatini had a national pharmacy policy, but not a drug policy. A standalone pharmacovigilance policy was enforced in Nigeria and Tanzania. Acts providing for the establishment of the NMRA were in place in all four countries. Overall, this assessment, which encompassed various indicators, highlighted that, while most countries had established laws, regulations, and guidelines for PV, the ability of national PV systems to effectively prevent adverse drug reactions (ADRs) remained limited due to several obstacles, including confusion about roles and responsibilities for the PV of new medicines and duplication of efforts (15).

Addressing these issues hinges on delivering targeted interventions to scale up training and mentoring of local HCPs and PV stakeholders that are part of the PAVIA project’s wider strategy to enhance post-marketing surveillance capacity in these countries.

Until recently, medical education of HCPs in resource-constrained SSA countries had mostly relied on traditional face-to-face didactic formulas. However, in last decade the need to scale-up an over-burdened and often inadequate educational infrastructure combined with a rapid and extensive spread of mobile technology in Africa have led to the broad adoption of novel learning approaches such as e-learning (16).

E-learning is defined as the use of information technology or Internet for learning activities. It is a flexible, time-saving, cost-saving approach that allows students to easily access contents from anywhere, at any time (17).

A blended e-learning training approach can be developed by integrating components from both traditional training methods and fully online learning, harnessing the strengths of each while overcoming their respective limitations (18). Several studies in the literature have underscored the efficacy of blended e-learning programs. The adoption of blended e-learning programs in resource-constrained SSA countries has resulted in notable cost savings and enhanced knowledge acquisition for training HCPs (19–24).

Combining a blended e-learning with a Training of Trainers (ToT) framework represents an effective way to scale up training of HCPs. This model involves initially training a group of learners to become trainers who can then impart their knowledge to multiple student groups. The ToT scheme is particularly well-suited for resource-constrained settings due to its cost-effectiveness and its ability to rapidly and comprehensively enhance the skills of HCPs (25, 26).

The present work aimed at evaluating the effectiveness of a blended e-learning-based ToT program comprising two courses designed to train HCPs on PV general principles and anti-TB drugs safety in the four PAVIA countries. Additionally, a comparison between the implementation of the program prior and after the beginning of the COVID-19 pandemic was made. The program was part of a broader strategy set up by PAVIA to promote PV in SSA countries.

## Materials and methods

### Development of a web-based application for the e-learning program

A web-based application named Schoolroom was developed by MedBrains, a spin-off born within the University of Verona.^2^ The specific aim was to provide tutors and learners with a user-friendly platform for easily managing their online classrooms and accessing e-learning materials, respectively. To improve content accessibility in settings where Internet connectivity is still limited or even totally absent, Schoolroom was adapted for both online and offline use. Thus, e-learning materials were made available either via web or through a specific desktop version of the application which was pre-installed on USB memory sticks that were subsequently distributed to tutors. An internet connection was only necessary for initial access to the e-learning courses and for sharing user’s results with tutors. After the initial access, users could complete both courses offline.

Thanks to its versatility, the web application could be used on various devices, including smartphones and personal computers, with compatibility depending on the device’s browser. For an optimal browsing experience, we recommended users to use Google Chrome on Android devices, Windows/Mac PCs, and Safari on iPhone and iPad devices.

Both versions of Schoolroom required users to sign in with their own credentials. Credentials were provided by tutors to learners at the end of each first session of the training. Upon logging in, users had the option to access various sections within the application or perform specific functions based on their assigned roles.

Schoolroom included three areas, namely (1) Users, (2) Statistics and (3) Courses areas. Users with appropriate permissions could register new users and assign them specific roles in the (1) Users area. Individual or clustered data on users learning progresses were displayed in the (2) Statistics area. Finally, the (3) Courses area served as an interactive area where learners could access all e-learning materials.

Schoolroom allowed five roles to be assigned to users, each one with different view/edit permissions and privileges: (1) Administrator, (2) Tutor, (3) Sub-Tutor, (4) Participant and (5) Observer. (1) Administrators could access all Schoolroom areas and assign Tutor role to designated users. (2) Tutors could enroll and assign users as Participants; they could also check Participants progresses in courses within the Statistics area at any time. (3) Sub-Tutors could create and manage their own classes of Participants. (4) Participants were allowed to access all e-learning materials within the Courses area but could not view content located in other areas. Finally, (5) Observers had limited access permissions and were only allowed to enter the Statistics area.

### The e-learning courses

Two e-learning courses were developed: “The Basic Concepts in Pharmacovigilance” course (Course 1) and “An Overview of Tuberculosis and Anti-Tuberculosis Drug Safety Issues, Monitoring and Management (aDSM)” course (Course 2). The two courses covered different topics related to passive and active surveillance from both a global and a local perspective. Specifically, the first course described the basic principles of PV (Supplementary Table 1), while the second course focused on anti-TB drugs and their management, active surveillance of anti-TB drugs (Supplementary Table 2). It was estimated that each course would take about 3 h to complete.

Each course included both a non-country-specific introductory part and an additional country-specific section. Each part was further subdivided into a subset of educational modules expressly designed in a self-study format.

Each educational module had objectives specific to the module, key-points to summarize the module contents, special sections called “Practical Approach” containing practical advice for the learner, color images and tables as needed, clickable links to external web sources, up-to-date, guidelines and a list of references. This division of the course content into smaller units was designed to expedite the learning process while ensuring that students gained a comprehensive understanding of the subject. Along with educational modules, the e-learning courses incorporated two interactive modules per course, each presenting a different clinical case-based assignment that learners were asked to solve.

To assess learners pre-existing and acquired post-course knowledge, a pre-test and a post-test were included at the beginning and at the end of each of the two e-learning courses. Both used 10 identical, multiple-choice questions randomly generated by the web-based application where e-learning materials were uploaded.

Furthermore, each module included its own intermediate test comprising two multiple-choice questions. These intermediate tests were obligatory, requiring learners to answer the questions to proceed to the next module.

### Implementing the train of trainers scheme

In order to expand the training of the local healthcare workforce and reach a maximum number of HCPs, we incorporated our blended e-learning package into a ToT scheme for the implementation in the four PAVIA countries. The underlying rationale for this decision was that a ToT-based blended e-learning program could equip the initial group of local HCPs with the skills and knowledge required to effectively train a second group, potentially setting off a training cascade. The HCPs initially trained were expected to assume the role of trainers in the second phase of the ToT scheme.

On this account, we established two distinct implementation levels or stages for our strategy:

- a first level that involved a multi-day pilot training program with a blended e-learning approach, specifically designed for an initial group of potential trainers;
- a second level that involved newly trained tutors (defined as sub-tutors) who would instruct multiple cohorts of HCPs through an extended version of the pilot training course.

While the initial plan was for all first-level pilot training programs to be conducted on-site by our team, this was only feasible in the case of Tanzania. Due to travel limitations imposed by the COVID-19 pandemic, making it impossible to travel to Africa, all other pilot training programs incorporated sessions delivered through a widely used videoconferencing platform.

### Assessment of the training program

We identified the following measures for assessing the effectiveness of our training program:

- The primary evaluation outcome was the knowledge gain resulting from the completion of the e-learning courses. This was assessed by measuring differences in learners’ post-test and pre-test scores, using a paired Student’s t-test.
- We also assessed learners’ satisfaction with the training package and potential changes in individual attitudes toward PV after the training. These were assessed using an online evaluation questionnaire (Supplementary Table 3) and an online attitude questionnaire (Supplementary Table 4), which were both administered to learners at the end of each training. Both questionnaires utilized a Likert scale to measure learners’ satisfaction with the training programme and attitude toward PV. The evaluation questionnaire comprised four multiple-choice grid questions designed to measure learners’ satisfaction with the usability of the e-learning platform, the e-learning content, and the teaching methodology. Additionally, two single-choice questions collected feedback from learners regarding the availability of an Internet connection during the training and the type of device used to access the e-learning content. The attitude questionnaire included five multiple-choice grid questions and a single-choice question, with a focus on assessing learners’ attitudes toward PV and the spontaneous reporting of ADRs.
- Finally, we performed a qualitative analysis of the Individual Case Safety Reports (ICSRs) that were entered into VigiBase, the WHO global database of ICSRs, by the National Pharmacovigilance Centers of the PAVIA countries before and after the pilot trainings. This analysis aimed to evaluate the potential impact of our strategy on the spontaneous reporting systems of these countries. Specifically, a comparison was made between the number and type of ICSRs (encompassing both drugs-related and vaccines-related ICSRs, including COVID-19 vaccines-related) entered into VigiBase 1 year before and 1 year after the pilot trainings. ICSRs of Tanzania, Eswatini, and Nigeria were downloaded from VigiBase and characterized in terms of ADRs seriousness and reporter qualification. The analysis of ICSRs entered into VigiBase by the Ethiopia National Pharmacovigilance Centre could not be performed due to the non-implementation of the step-down trainings, stemming from organizational hurdles, which led to the creation of a limited pool of potential reporters.

Further, we computed the percentage of ICSRs associated with anti-TB drugs based solely on the number of ICSRs that were linked to drug treatments.

All data related to the ICSRs of interest were obtained via VigiLyze, the web-based ICSRs data management system managed by the WHO Uppsala Monitoring Centre (27).

## Results

We conducted the PAVIA pilot training in Dar Es Salaam, Tanzania, in October 2019 (Supplementary Table 5). As the sole pilot training conducted before the onset of the COVID-19 pandemic, this training was also the only one to incorporate face-to-face sessions. A total of 36 learners attended the 5-days blended e-learning-based training, which combined face-to-face sessions delivered by three Italian tutors and one local tutor and e-learning-based sessions during which learners were required to go through the two PAVIA e-learning courses. All learners (*n* = 36) passed the post-tests at the end of each course. Throughout the course, participants interacted with each other and the local coordinators, posing targeted questions to the tutors about the content. As part of a test simulating the implementation of the ToT scheme, all participants successfully enrolled students in the Schoolroom platform. Additionally, a PV comedy (performed in Swahili) was created, where two students humorously portrayed a scenario in which a patient reported an ADR to his/her general practitioner.

Similarly, a multi-day pilot training program was implemented and adopted in all other countries.

In April 2021, the Eswatini pilot training was conducted remotely due to international travel restrictions imposed worldwide during the COVID-19 pandemic. In-person sessions were replaced with virtual sessions that were delivered via a videoconferencing platform. Similarly, pilot trainings aimed at Nigeria and Ethiopia were delivered remotely in January 2022 and July 2022, respectively. 34, 25, and 26 learners enrolled in the pilot trainings conducted in Eswatini, Nigeria, and Ethiopia, respectively.

Except for 2 out of 26 learners who participated in the pilot training for Ethiopia and did not complete the post-tests after completing the e-learning courses, all other participants successfully finished the training. We found that learners’ scores on the post-tests were higher than on the pre-tests for both courses. Upon applying the Student’s t-test to pre-test and post-test’s results, a positive significant difference was found in Tanzania, Eswatini and Nigeria (Table 1). The *p*-value for the paired t-test for the difference in mean score on the pre-and post-test for each module was <0.001.

**Table 1:** Main features and results of the PAVIA pilot trainings conducted in the four PAVIA countries (e-learning course 1: “The Basic Concepts in Pharmacovigilance”; e-learning course 2: “An Overview of Tuberculosis and Anti-Tuberculosis Drug Safety Issues, Monitoring and Management (aDSM)”).

PAVIA country	N. of learners registered in Schoolroom	N. of learners who attended the pilot training and successfully completed the training program (%)	Time period	N. of days	Program structure	Post-test vs. pre-test Student’s *t* test score
Tanzania	36	36	October 2019	5	Face-to-face and online e-learning sessions*	E-learning course 1: 13.32 (*p* < 0.00001)
						E-learning course 2: 11.59 (*p* < 0.00001)
Eswatini	34	34	April 2021	4	Virtual sessions and online e-learning sessions	E-learning course 1: 7.91 (*p* < 0.00001)
						E-learning course 2: 10.77 (*p* < 0.00001)
Nigeria	25	25	January 2022	4	Virtual sessions and online e-learning sessions	E-learning course 1: 5.03 (*p* < 0.00001)
						E-learning course 2: 9.02 (*p* < 0.00001)
Ethiopia	26	26	July 2022	4	Virtual sessions and online e-learning sessions	E-learning course 1: 4.96 (*p* < 0.00001)
						E-learning course 2: 7.93 (*p* < 0.00001)
Total	121	121 (100%)	-	-	-	-

In the years following the implementation of the pilot trainings, multiple step-down training sessions were delivered in Tanzania, Eswatini, and Nigeria by 39 sub-tutors, including 17 in Tanzania, 14 in Eswatini and 8 in Nigeria, thus successfully implementing the second level of the program in these countries. Out of the 827 learners that were registered in Schoolroom by tutors, including 319 learners in Tanzania, 375 learners in Eswatini and 133 learners in Nigeria, 472 attended the step-down trainings. Of these, 421 learners (89%) successfully completed the training program, including 157 learners in Tanzania, 178 learners in Eswatini and 86 learners in Nigeria. It is worth noting that 37 learners in Nigeria completed only course 1 as they attended a shorter version of the training package that included only the first course. This decision was based on their professional background and qualifications, which did not relate to the management of anti-TB drugs. The remaining 49 learners did both the courses.

We found that learners’ scores on the post-tests were higher than on the pre-tests for both courses. Upon applying the Student’s t-test to pre-test and post-test’s results, a positive significant difference was found in Tanzania, Eswatini and Nigeria (Table 2). Again, the *p*-value for the paired t-test for the difference in mean score on the pre- and post-test for each module was <0.001. Unlike the other three countries, Ethiopia could not implement the step-down trainings because of organizational hurdles.

**Table 2:** Main features and results of the PAVIA step-down trainings conducted in Tanzania, Eswatini and Nigeria (e-learning course 1: “The Basic Concepts in Pharmacovigilance”; e-learning course 2: “An Overview of Tuberculosis and Anti-Tuberculosis Drug Safety Issues, Monitoring and Management (aDSM)”).

PAVIA country (N. of sub-tutors)	N. of learners registered in Schoolroom	N. of learners who attended the step-down trainings (%)	N. of learners who successfully completed the training program on the number of attendees (%)	Post-test vs. pre-test Student’s *t* test score
Tanzania (17)	319	175 (55%)	157 (90%)	E-learning course 1: 16.30 (*p* < 0.00001)
				E-learning course 2: 16.77 (*p* < 0.00001)
Eswatini (14)	375	200 (53%)	178 (% 89%)	E-learning course 1: 7.37 (*p* < 0.00001)
				E-learning course 2: 9.51 (*p* < 0.00001)
Nigeria (8)	133	97 (73%)	86^*^ (%89%)	E-learning course 1: 13.01 (*p* < 0.00001)
				E-learning course 2: 9.65 (*p* < 0.00001)
Total	827	472 (57)	421 (89%°°)	-

^*^37/86 learners who attended the step-down trainings in Nigeria only did course 1 as they undertook a shorter version of the training package that included only the first course due the fact that their professional background and professional qualification did not related to the management of anti-TB drugs.

Most of those who attended the pilot trainings in the four PAVIA countries completed the evaluation and the attitude questionnaires (Table 3). Specifically, 111 out of 121 learners (92%) who attended the pilot trainings provided feedback on the training program: 35 in Tanzania (97%), 34 in Eswatini (100%), 22 in Nigeria (88%), and 20 in Ethiopia (77%). The overall feedback was positive, with 107 out of 111 respondents (96%) expressing satisfaction to strong satisfaction with the pilot training program. A majority of respondents, 93 out of 111 (84%), reported that Internet connection was at least available, albeit sometimes limited, during the training. 111 out of the 121 learners (92%) who attended the pilot trainings completed the attitude questionnaires. These included 36 out of 36 learners in Tanzania (100%), 31 out of 34 in Eswatini (91%), 22 out of 25 in Nigeria (88%), and 22 out of 26 in Ethiopia (85%). Remarkably, 110 out of 111 learners (99%) reported an improved perception of PV after the training, indicating a significant positive shift in their attitudes toward PV. The stark majority, as indicated by 102 out of 111 respondents (92%), stated that they had submitted or were willing to submit at least one ADR report to local PV centers after attending the training (Table 4).

**Table 3:** Distribution of respondents providing feedback via evaluation and attitude questionnaires.

PAVIA country	Evaluation questionnaire		Attitude questionnaire	
	N. of respondents/N. of learners who completed the training (%)		N. of respondents/N. of learners who completed the training (%)	
	Pilot training	Step-down trainings	Pilot training	Step-down trainings
Tanzania	35/36 (97%)	44/157 (28%)	36/36 (100%)	34/157 (22%)
Eswatini	34/34 (100%)	9/178 (5%)	31/34 (91%)	12/178 (7%)
Nigeria	22/25 (88%)	63/86 (73%)	22/25 (88%)	59/86 (69%)
Ethiopia	20/26 (77%)	-	22/26 (85%)	-
Total	111/121 (92%)	116/421 (28%)	111/121 (92%)	105/421 (25%)

**Table 4:** Summary of key findings from evaluation and attitude questionnaires.

PAVIA country	High satisfaction with PAVIA training program		Reliable Internet connectivity during the training		Improved perception of pharmacovigilance after the training		Willingness to report at least an ADR after the training	
	N. of positive responses/Total respondents		N. of positive responses/Total respondents		N. of positive responses/Total respondents		N. of positive responses/Total respondents	
	(% of positive responses)		(% of positive responses)		(% of positive responses)		(% of positive responses)	
	Pilot training	Step-down trainings	Pilot training	Step-down trainings	Pilot training	Step-down trainings	Pilot training	Step-down trainings
Tanzania	32/35 (91%)	41/44 (93%)	24/35 (69%)	36/44 (82%)	36/36 (100%)	32/34 (94%)	28/36 (78%)	19/34 (56%)
Eswatini	34/34 (100%)	8/9 (89%)	30/34 (88%)	7/9 (78%)	30/31 (97%)	12/12 (100%)	30/31 (97%)	12/12 (100%)
Nigeria	22/22 (100%)	63/63 (100%)	19/22 (86%)	60/63 (95%)	22/22 (100%)	59/59 (100%)	22/22 (100%)	57/59 (97%)
Ethiopia	19/20 (95%)	-	20/20 (100%)	-	22/22 (100%)	-	22/26 (85%)	-
Total	107/111 (96%)	112/116 (97%)	93/111 (84%)	103/116 (89%)	110/111 (99%)	103/105 (98%)	102/111 (92%)	88/105 (84%)

Only 116 out of 421 learners (28%) who attended the step-down trainings provided feedback on the training program: 44 out of 157 in Tanzania (28%), 9 out of 178 in Eswatini (5%), and 63 out of 86 in Nigeria (73%). Despite a response rate lower than the one recorded after the pilot trainings, 112 out of 116 respondents (97%) reported strong to very strong satisfaction with the step-down trainings. Respondents who said that Internet connection was available were 103 (89%). The number of those who attended the step-down trainings and completed the attitude questionnaire was 105 (25%), including 34 respondents in Tanzania (22%), 12 in Eswatini (7%) and 59 in Nigeria (69%). Of these, 103 (98%) said that they felt more involved in PV after attending the training. Moreover, 88 out of 105 respondents (84%) confirmed that they had submitted or were willing to submit at least one ADR report to local PV centers after the training (Table 4).

In order to assess the potential impact of the blended e-learning-based PAVIA strategy on the PAVIA countries spontaneous reporting systems, a qualitative analysis of the ICSRs entered in VigiBase by the National Pharmacovigilance Centres of three out of four PAVIA countries was performed (Table 5).

**Table 5:** Number and features of ICSRs entered in VigiBase 12 months prior and after the pilot trainings in Tanzania, Eswatini and Nigeria.

	Tanzania		Eswatini		Nigeria	
	Pre-pilot training (% of vaccines-related ICSRs, % of COVID-19 vaccines-related ICSR)	Post-pilot training (% of vaccines-related ICSRs, % of COVID-19 vaccines-related ICSR)	Pre-pilot training (% of vaccines-related ICSRs, % of COVID-19 vaccines-related ICSR)	Post-pilot training (% of vaccines-related ICSRs, % of COVID-19 vaccines-related ICSR)	Pre-pilot training (% of vaccines-related ICSRs, % of COVID-19 vaccines-related ICSR)	Post-pilot training (% of vaccines-related ICSRs, % of COVID-19 vaccines-related ICSR)
Total n. of ICSRs	2,369 (0–0%)	14,512 (0–0%)	717 (0–0%)	1,222 (86–86%)	13,284 (70–67%)	15,986 (86–76%)
N. of ICSRs concerning serious ADRs (%)	315 (13%) (0–0%)	197 (1%) (0–0%)	124 (17%) (1–0%)	74 (6%) (66–66%)	1,675 (13%) (59–45%)	2,230 (14%) (72–40%)
N. of anti-TB drugs-related ICSRs (%)^*^	64 (3%)	142 (1%)	285 (40%)	67 (40%)	419 (11%)	175 (8%)
Most prolific reporters	Pharmacists (2,194)	Pharmacists (14,010)	Physicians (460)	Other HCPs (637)	Other HCPs (9,744)	Other HCPs (14,346)

^*^The percentage of ICSRs associated with anti-TB drugs was calculated based solely on the number of ICSRs linked to drug treatments.

ICSRs entered into VigiBase by the National Pharmacovigilance Centre of Tanzania over a one-year period before the first PAVIA training, that is, from 25.10.2018 to 25.10.2019, and over a one-year period after it, from 25 October 2019 to 26 October 2020, were retrieved via VigiLyze. Data analysis revealed that 2,369 ICSRs were received before the training and 14,512 ICSRs were received after, marking a net increase of 12,143 reports (+513%). Of the 2,369 ICSRs received before the pilot training, 2,022 concerned non-serious ADRs, 315 concerned serious ADRs and 32 concerned ADRs of unknown seriousness. At that time, the most prolific reporters were pharmacists, who submitted 2,194 ICSRs.

Of the 14,512 ICSRs received after the pilot training, 14,315 concerned non-serious ADRs and 197 concerned serious ADRs. No ICSRs with ADRs of unknown seriousness were received. The most prolific reporters remained pharmacists, who submitted 14,010 ICSRs. While the absolute number of ICSRs associated with anti-TB drugs increased from 64 before the pilot training to 142 after the pilot training, the percentage of these reports relative to the total decreased from 35 to 1%. No ICSRs associated with vaccines were received before or after the training.

ICSRs entered into VigiBase by the National Pharmacovigilance Centre of Eswatini over a one-year period before the first PAVIA training, that is, from 29 April 2020 to 29 April 2021, and over a one-year period after it, from 30 April 2021 to 30 April 2022, were retrieved via VigiLyze. Data analysis revealed that 717 ICSRs were received before the training and 1,222 ICSRs were received after, marking an increase of 505 reports (+70%). Of the 717 ICSRs received before the pilot training, 593 concerned non-serious ADRs and 124 concerned serious ADRs. The most prolific reporters were physicians, who submitted 460 ICSRs.

Of the 1,222 ICSRs received after the pilot training, 1,148 concerned non-serious ADRs and 74 concerned serious ADRs. No ICSRs with ADRs of unknown seriousness were received. The most prolific reporters were other HCPs, who submitted 637 ICSRs. After the training, there was a significant decrease in the absolute number of ICSRs associated with anti-TB drugs, decreasing from 285 to 67, while the percentage remained unchanged at 40%. On the other hand, before the pilot training, there were hardly any reports related to vaccines. However, following the training, there was a significant increase in these reports, jumping from 0% to 86%. Notably, all the reported vaccines were for COVID-19.

ICSRs entered into VigiBase by the National Pharmacovigilance Centre of Nigeria over a one-year period before the first PAVIA training, that is, from 20 January 2021 to 20 January 2022, and over a one-year period after it, from 21 January 2022 to 21 January 2023, were retrieved via VigiLyze. Data analysis revealed that 13,284 ICSRs were received before and 15,986 ICSRs after the pilot training, marking a slight increase of 2,702 reports (+20%). Of the 13,284 ICSRs received before the pilot training, 11,609 concerned non-serious ADRs and 1,675 concerned serious ADRs. The most prolific reporters were other HCPs, who submitted 9,774 ICSRs.

Of the 15,986 ICSRs received after the pilot training, 13,756 concerned non-serious ADRs and 2,230 concerned serious ADRs. No ICSRs with ADRs of unknown seriousness were received. The most prolific reporters were other HCPs, who submitted 14,346 ICSRs. After the training, there was a significant decrease in both the absolute number and the percentage of ICSRs related to anti-TB drugs, dropping from 419 to 175 and from 115 to 8%, respectively. The proportion of ICSRs related to vaccines remained high both before the pilot training (70%) and after (86%). Moreover, the majority of these ICSRs consisted of COVID-19 vaccine reports, comprising 67% before the pilot training and 76% after the pilot training.

We did not assess the quality of ICSRs, as such an analysis would have necessitated a longer observation period.

## Discussion

The present work represents an example of an innovative strategy specifically designed to strengthen PV in four African countries through a blended e-learning-based ToT program. As far as we know, even though some training on PV had been previously provided on the ground by the African partners, this was one of the first educational experience involving fully remote training on PV to be conducted between Italy and multiple African countries via a videoconferencing platform. We demonstrated the effectiveness and the adaptability of an innovative approach that brings together elements of the blended e-learning methodology, the ToT model, and the use of video conferencing tools to mentor HCPs in both PV and the safety of anti-TB drugs.

The implementation of the second level of our strategy was carried out by those learners who had been trained in the first stage of the program. Those newly trained tutors conducted multiple step-down trainings under the supervision of PAVIA local coordinators. Moreover, our team was able to monitor the progress of single individuals and entire classrooms and provided technical assistance at tutors’ request. Out of the 827 learners registered by local tutors in Schoolroom to participate in the step-down trainings conducted in Tanzania, Nigeria, and Ethiopia, 421 (51%) completed the program and received certificates. One possible explanation for this outcome is that, after being registered on the e-platform by tutors, some learners were unable to attend due to various reasons such as lack of connectivity, difficulty accessing the training site, administrative hurdles like permissions and reimbursement issues, among other challenges. Out of 472 who enrolled in the step-down trainings, 421 (89%) completed the training program. The number of successful learners in Nigeria included those learners who attended a shorter version of the training program based on the sole completion of Course 1. Distance and blended e-learning approaches in the field of pharmacovigilance are not novel concepts. Several studies in the literature have underscored the effectiveness of blended e-learning programs in healthcare, including pharmacovigilance (19–24). For instance, Rudd et al. conducted an evaluation of a blended e-learning course in Namibia and Tanzania, concluding that this approach is an effective method for training healthcare workers in the fundamental features of electronic health information systems (19).

Unlike in the other three countries, no step-down trainings were conducted in Ethiopia due to unspecified organizational hurdles.

More than 90% of those who attended the pilot trainings in the four PAVIA countries completed and submitted both the evaluation and the attitude questionnaires. Learners that attended the pilot trainings were satisfied with the contents and structure of the courses and, in general, with how the blended e-learning program was implemented in each country. No significant difference in terms of appreciation between learners who attended the in-person sessions included into the pilot training for Tanzania and those who participated in the virtual sessions of the pilot trainings for Eswatini, Nigeria, and Ethiopia was observed.

Measuring satisfaction of those learners who attended the step-down trainings proved difficult, as most of them (73%) did not complete and submit the evaluation questionnaire. As in the case of evaluation questionnaires, attitude questionnaires were not submitted by most of these learners (75%), thus strongly limiting the assessment of learners’ perception of PV after the training. While being low, the response rate is consistent with similar trends reported in the literature (28).

Still, in both cases the limited data that we managed to collect suggest that even those learners who attended step-down trainings were satisfied with the training experience and that their interest in PV increased after the training. In particular, a significant number of respondents submitted or were willing to submit at least an ADR report after the training. As new tutors were left free to set up trainings in accordance with their own, any possible feedback provided by learners might have been affected by the individual decisions taken by local tutors regarding the duration and the preferred mode of implementation of the training. Additionally, organizational hurdles related to the distribution of questionnaires to students could have impacted the reliability of these results. Future research should address these factors for a more comprehensive understanding.

Replacing the in-person sessions with virtual sessions was the most feasible way to overcome the challenge posed by the inability to travel abroad during the COVID-19 pandemic. We chose to use a well-known videoconferencing platform because of its widespread availability and usability. This turned out to be wise choice as the use of a free version of the platform allowed us to reach learners without incurring in major technical issues. Adopting virtual meetings as teaching tool came with both advantages and limitations.

This could be seen clearly during the implementation of the first pilot training program in Tanzania, which involved more in-person dialog, conversations and real time interaction between learners than the other pilot trainings. Learners seemed at ease in a familiar, traditional classroom setting without technological barriers. The in-person interaction facilitated a more personal connection with learners, enhancing the exchange of information. On the other hand, Kiguli-Malwadde et al., who assessed the impact of transitioning a multi-country HIV training program from in-person to online, compared digital training approaches implemented during the pandemic with in-person approaches used before COVID-19. Their study concluded that participants in in-person learning programs exhibited greater gains in knowledge and clinical confidence than those engaged in online learning (29).

In this training section, all participants were engaged and enthusiastic; they demonstrated a keen interest in learning the program and a strong desire to broaden their knowledge on the presented topics. Questions from the audience were precise and relevant. Notably, on day 4, participants exhibited remarkable effort when tasked to take on the role of “trainers.” The inclusion of a ‘pharmacovigilance comedy’ played a pivotal role at the session’s conclusion, proving to be a success as participants interpreted it with the right spirit.

At the same time, relying on remote sessions to reach out to learners during COVID-19 pandemic demonstrated that, while the lack of face-to-face contact made difficult to interact with learners sometimes, it cut costs and time. Rudd et al. evaluated a blended e-learning course in Namibia and Tanzania reported that the cost is up to 3.4 times less expensive than for an in-person course with similar content (19). In addition, remote sessions allowed tutors from those PAVIA countries where the training program was at a more advanced stage to attend the trainings and share their own experience with conducting step-down trainings in their own country. This was strongly appreciated by learners, who had the opportunity to listen to and benefit from the experience of those who had already conducted second-level trainings in their own countries. The learning environment was responsive and engaging and attention level was always very high among the participants during all 3 days without any distraction. The greatest limitation of virtual meetings came from the poor Internet connectivity, which might have negatively impacted the learning experience sometimes. Although neither we nor learners experienced any meaningful technical issues during the virtual sessions, Internet connection was not always stable throughout the meetings, as many learners who attended different sessions reported in the evaluation questionnaire.

The lack of good connection or even Internet access might pose a significant challenge not only for tutors that choose to replace in person sessions with virtual meetings but also for those learners who live and work in remote areas of the countries of interest, who might find themselves unable to access the e-learning materials. To bypass this issue, we provided the newly appointed tutors of each PAVIA country with USB flash drives containing an offline version of the courses. These USB flash drives were effectively used in Tanzania, Eswatini and Nigeria to assist those learners who struggled to access the online contents because of poor connectivity.

The implementation of the PAVIA training program in multiple settings, though under similar conditions, offered an opportunity to test the functioning of Schoolroom, our e-learning platform. While most of learners used a computer rather than a smartphone to access Schoolroom, no significant technical issues were reported by both groups. This strongly argues in favor of the versatility of the platform and suggests that relying on a software that is both a desktop and a mobile-friendly platform might allow a widespread access to e-learning contents in settings where smartphones represent the most used device. Overall, Schoolroom was seen as a user-friendly, well-performing platform which most of learners were able to access to and navigate through.

Assessing the real impact of the pilot trainings on the spontaneous reporting systems of Tanzania, Eswatini, and Nigeria was not an easy task as we could not match reporters to learners who attended the trainings. In the year after the pilot trainings a net increase in the number of ICSRs entered into VigiBase by the national pharmacovigilance centers was observed in three countries out of four PAVIA countries, suggesting that training actually had a positive effect on reporting rate. With more than 14,000 ICSRs entered in the year following the pilot training, Tanzania was the country that registered the highest increase. Importantly, the increase in the number of ICSRs observed in Tanzania was not driven by reporting of vaccines-related ADRs.

Pharmacists submitted the highest number of both pre-training ICSRs and post-training ICSRs, persisting as the most active reporters in the country. The increase in the number of ICSRs followed a slightly different pattern in Eswatini and Nigeria, as it appeared to be much more limited Additionally, in both countries this increase in reporting rate was connected to active surveillance of COVID-19 vaccines, which were widely deployed in both countries in the last 2 years. The development and distribution of COVID-19 vaccines in 2020 and 2021, respectively, align with the observed trend in vaccination in these nations. The high number of COVID-19 vaccines-related ICSRs entered into VigiBase over the past few years made hard to tell for sure whether those numbers could be ascribed to spontaneous reporting or rather depended on the close monitoring of the safety of COVID-19 vaccines.

The percentage of ICSRs related serious ADRs decreased in the year following the pilot training in all countries except Nigeria, where a constant trend was observed. Numerous factors could have influenced the reporting trends, particularly with the emergence of the COVID-19 pandemic in 2020, which led to considerable disruptions in the health and pharmacovigilance systems of these countries. After the pilot training, Tanzania experienced a substantial increase in the total number of ICSRs, which might have contributed to a decrease in the proportion of serious ICSRs. Eswatini was the country most influenced by the COVID-19 vaccination trend in reporting, as our training took place in April 2021. The potential reason for the decrease of serious ICSRs in Eswatini could be attributed to the increase in non-serious ICSRs, mainly due to COVID-19 vaccine reports, resulting in a decreased proportion of ICSRs concerning serious ADRs. In other countries, a similar trend emerged. The Italian Medicines Agency released a report on COVID-19 vaccine safety, covering the initial 2 years of vaccine surveillance. The vast majority of vaccine-related ICSRs logged in the Italian pharmacovigilance database during this period were associated with non-serious events.

In Nigeria, we observed a consistent trend, where the influence of COVID-19 vaccination on reporting remained steady both before and after our training.

Considering this, incorporating an intermediate evaluation, such as 6 months after the intervention, could have provided a more comprehensive assessment of the data.

Across the three PAVIA countries, the analysis of ICSRs related to anti-TB drugs shows divergent trends. In Tanzania, there was an increase in the absolute number of those ICSRs, accompanied by a decrease in the percentage. In Eswatini, the absolute number decreased, while the percentage remained constant. Conversely, in Nigeria, both the absolute number and the percentage decreased. Due to the limited number of ICSRs associated with anti-TB drugs, it is difficult to provide a unified explanation for all PAVIA countries. In summary, we have designed and successfully implemented an innovative, blended e-learning program in the four PAVIA countries to train an extensive number of HCPs in both the basic principles of PV and the safety of anti-TB drugs. Over a span of more than 3 years, dedicated groups of newly-formed trainers conducted multiple step-down trainings in three of the four countries, training hundreds of HCPs and contributing to foster a culture of PV and spontaneous reporting in each country. Analysis of pre-test and post-test scores revealed that our e-learning courses effectively improved learners’ knowledge on the targeted topics, as significant learning progress was consistently observed following each training session. Overall, learners’ feedback on the training experience was positive, with most of them expressing satisfaction with the training and an increased interest in pharmacovigilance after their participation. The utilization of virtual sessions did not appear to affect learning outcomes, as there were any major differences between those learners who attended in-person sessions during the pilot training in Tanzania and those who received remote training. While remote training may have limited interaction between trainers and learners, it provided a vital avenue to reach a broader audience at a time when international travel was severely restricted due to the COVID-19 pandemic, making in-person training nearly impossible. To sum up, our experience shows that, despite its limitations, remote learning represents a versatile, cost-effective alternative to in person training and should be taken in consideration when planning for training in distant or less accessible areas. Schoolroom, our e-learning platform, performed well. No major technical issue arose during the trainings apart from poor connectivity. This was addressed by using USB flash drives and our platform, which could operate both online and offline. Similar projects should consider connectivity issues as a key factor. In conclusion, even when external conditions required us to conduct the training programs exclusively online in Eswatini, Nigeria, and Ethiopia, the system proved to be effective, yielding results comparable to those achieved through traditional methods. Finally, it is important to note that, while the trainings and other PAVIA initiatives might have had an impact on reporting rates in Tanzania, Eswatini, and Nigeria, a direct causal relationship between the trainings and the significant increase in ICSRs during the year following the pilot trainings could not be definitively established. This limitation arose because we were unable to match reporters to learners who attended the trainings. Additionally, it is crucial to acknowledge that the active surveillance of COVID-19 vaccines played a significant role in the observed upswing in the reporting rate.

## Author’s note

The information presented in this publication is sourced from VigiBase, the WHO global database of suspected adverse reactions to medicinal products, developed and maintained by Uppsala Monitoring Centre. It is important to note that the VigiBase data originates from various sources, and the likelihood of a suspected adverse effect being drug-related may vary across cases. Furthermore, the opinions expressed herein do not reflect those of the Uppsala Monitoring Centre or the World Health Organization.

## Data availability statement

The dataset is not available due to the fact that it contains participants identifiable data that cannot be made public.

## Author contributions

FS: Conceptualization, Writing – original draft, Writing – review & editing. KM: Writing – review & editing. SK: Writing – review & editing. EM: Writing – review & editing. AD: Writing – review & editing. SN: Writing – review & editing. CE: Writing – review & editing. AA: Writing – review & editing. SM: Writing – review & editing. SP: Data curation, Writing – review & editing. RL: Methodology, Software, Writing – review & editing. AS: Methodology, Software, Writing – review & editing. FC: Project administration, Writing – review & editing. LH: Project administration, Writing – review & editing. EE: Writing – review & editing. AC: Writing – review & editing. MV: Conceptualization, Supervision, Writing – review & editing. LM: Conceptualization, Supervision, Writing – review & editing. UM: Writing – review & editing, Conceptualization, Supervision.

## Footnotes

^1^ https://pavia-africa.net/

^2^ https://www.medbrains.it/en/
